# Early Restenosis of Sirolimus-eluting Stent: An Unusual Case of a Hyperthyroid Patient

**DOI:** 10.7759/cureus.6490

**Published:** 2019-12-28

**Authors:** Syeda Javeria Shabbir, Mariam Baloch, Faryal Mustafa, Hira Maab, Laila Tul Qadar

**Affiliations:** 1 Internal Medicine, Dow University of Health Sciences, Karachi, PAK; 2 Medicine, Dow Medical College, Dow University of Health Sciences, Karachi, PAK

**Keywords:** hyperthyroidism, drug-eluting stents, multinodular goiter, cardiac catheterization, coronary stents, stent thrombosis, in-stent restenosis

## Abstract

The presentation of atherosclerosis with concomitant hyperthyroidism is not uncommon. Hyperthyroidism predisposes to worse cardiovascular pathologies like systolic hypertension, atrial fibrillation, and hypercoagulability. Drug-eluting stents, on the other hand, have emerged as a miracle treatment choice for patients having atherogenic conditions. They have the highest success rates when it comes to minimizing in-stent restenosis (ISR) during short-term follow-up. There is scarce literature that assesses the correlation of multinodular goiter (MNG) to ISR, especially in Pakistan, and thus any probable association between the two is left untouched. We report a case of a 57-year-old female who is a known hyperthyroid with a massive MNG, presenting with worsening chest pain. She had undergone sirolimus-eluting stent (SES) implantation in left anterior descending artery (LAD) six months back. Cardiac catheterization confirmed restenosis of the SES in the LAD, along with the occlusion of left circumflex and right coronary artery, accompanied by grade I diastolic dysfunction and mild aortic regurgitation on echocardiography.

## Introduction

Hyperthyroidism is defined as excessive T3 production due to thyroid gland hyperfunctioning. Peripheral monodeiodination of T4 is increased, which ultimately results in reflective changes in the cardiovascular system [1]. A fraction of patients having thyroid disease have cardiac disease, either coronary or valvular, as a common finding [2]. In exceptional cases, hyperthyroid patients can present with chest pain and echocardiography changes of cardiac ischemia [3]. In elderly patients with coronary artery disease (CAD), an increase in cardiac contractility and workload associated with hyperthyroidism reveals an increase in myocardial oxygen demand [1]. There is a higher risk of CAD and increased death rate associated with overt and subclinical hyperthyroidism [4]. Hyperthyroidism has been related to an increased activity of factor X in coagulation cascade which is simultaneously related to increased risk of CAD [5]. Restenosis is defined as a decrease in the luminal diameter of more than 50% after percutaneous coronary intervention, either with or without stent implantation. Interventional cardiologists have always considered in-stent restenosis (ISR) to be the main culprit, and hence many techniques have been introduced in the last 20 years to reduce its incidence: first newer generation bare-metal stents (BMS), then drug-eluting stents (DES), and finally drug-coated balloons [6]. With the arrival of DES the incidence of restenosis has reduced to numbers <10% [7]. Here, we present the case of 57-year-old female, a known hyperthyroid, brought to the hospital with acute chest pain which was later established as secondary to the restenosis of sirolimus-eluting stent (SES) in the left anterior descending artery (LAD).

## Case presentation

**Figure 1:**
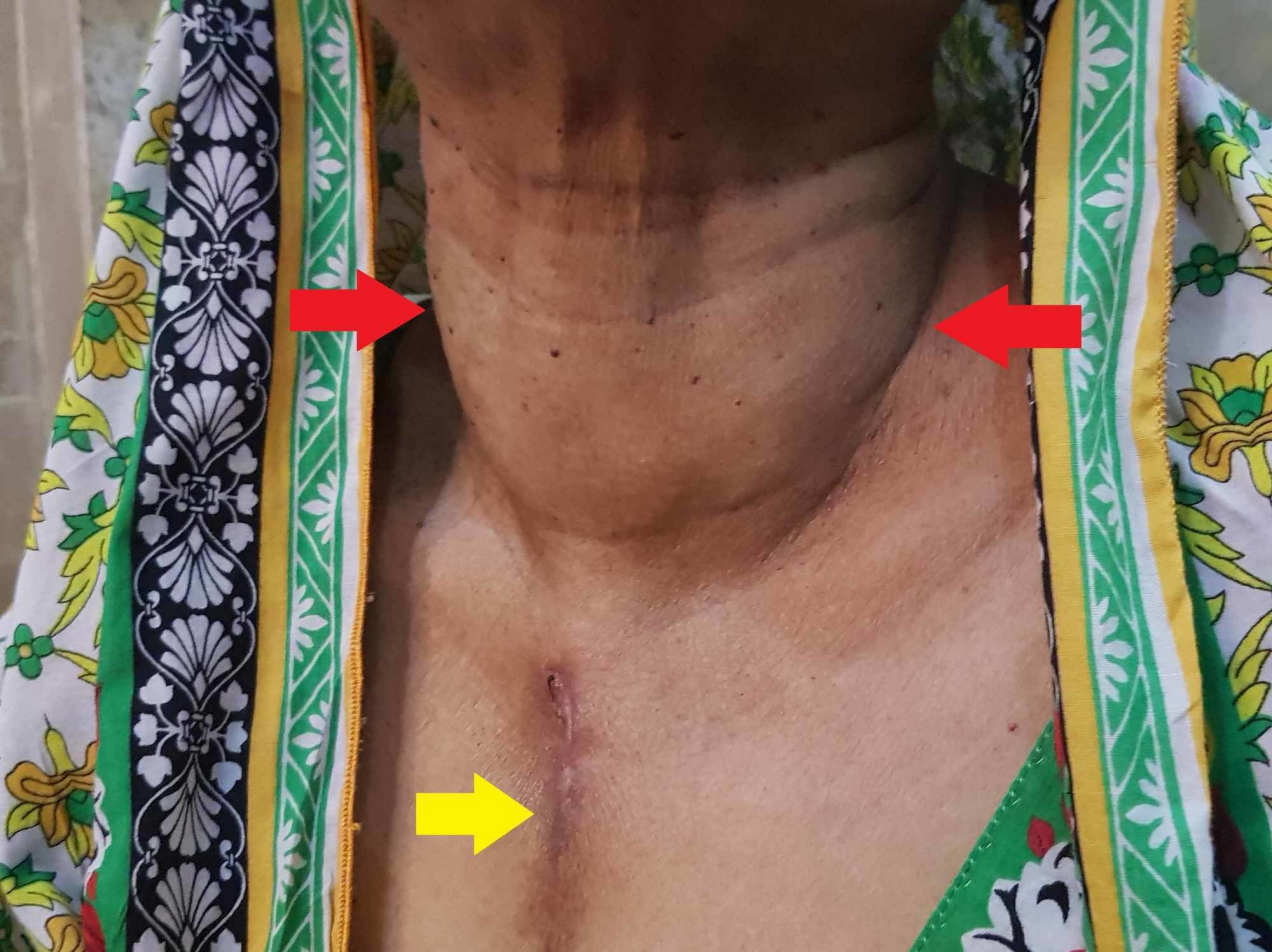
Visible goiter (red arrows) along with a clear previous median sternotomy incision scar (yellow arrow)

A 57-year-old female patient presented with gradual and progressive chest pain (Canadian Cardiovascular Society Grade III). Her past medical history included diabetes mellitus, hypertension, and hyperthyroidism. Previous surgical procedures included angioplasty where sirolimus-coated DES of 2.5 x 27 and 2.5 x 19 was placed in the LAD six months back. She had been taking neomercazole 5 mg for hyperthyroidism, glimepiride 2 mg for diabetes, and 160 mg valsartan for hypertension. On inspection, a prominent goiter was seen on her neck that moved with swallowing along with a median sternotomy scar, which indicated her previous thoracic surgery (Figure 1). 

**Figure 2:**
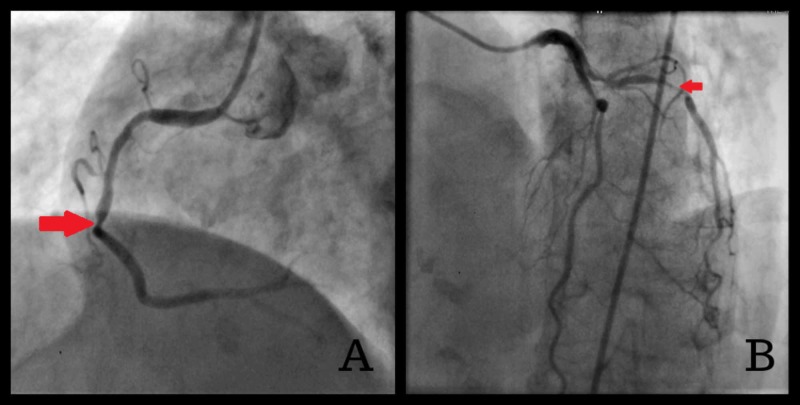
Coronary CT angiography images on admission (A) marked occlusion of mid-RCA (B) in-stent restenosis of the LAD CT, computed tomography; RCA, right coronary artery; LAD, left anterior descending artery.

On admission, the patient underwent cardiac catheterization which revealed the following findings: left main stem was normal, left circumflex artery was 90% occluded from proximal to mid with 80% disease in obtuse marginal 1. Right coronary artery was also diffusely diseased with 70%-80% mid vessel disease (Figure 2A). LAD also showed 40%-50% proximal diffuse stenosis, followed by ectatic segment with ISR in the mid to distal LAD-ectactic segment (Figure 2B).

**Figure 3:**
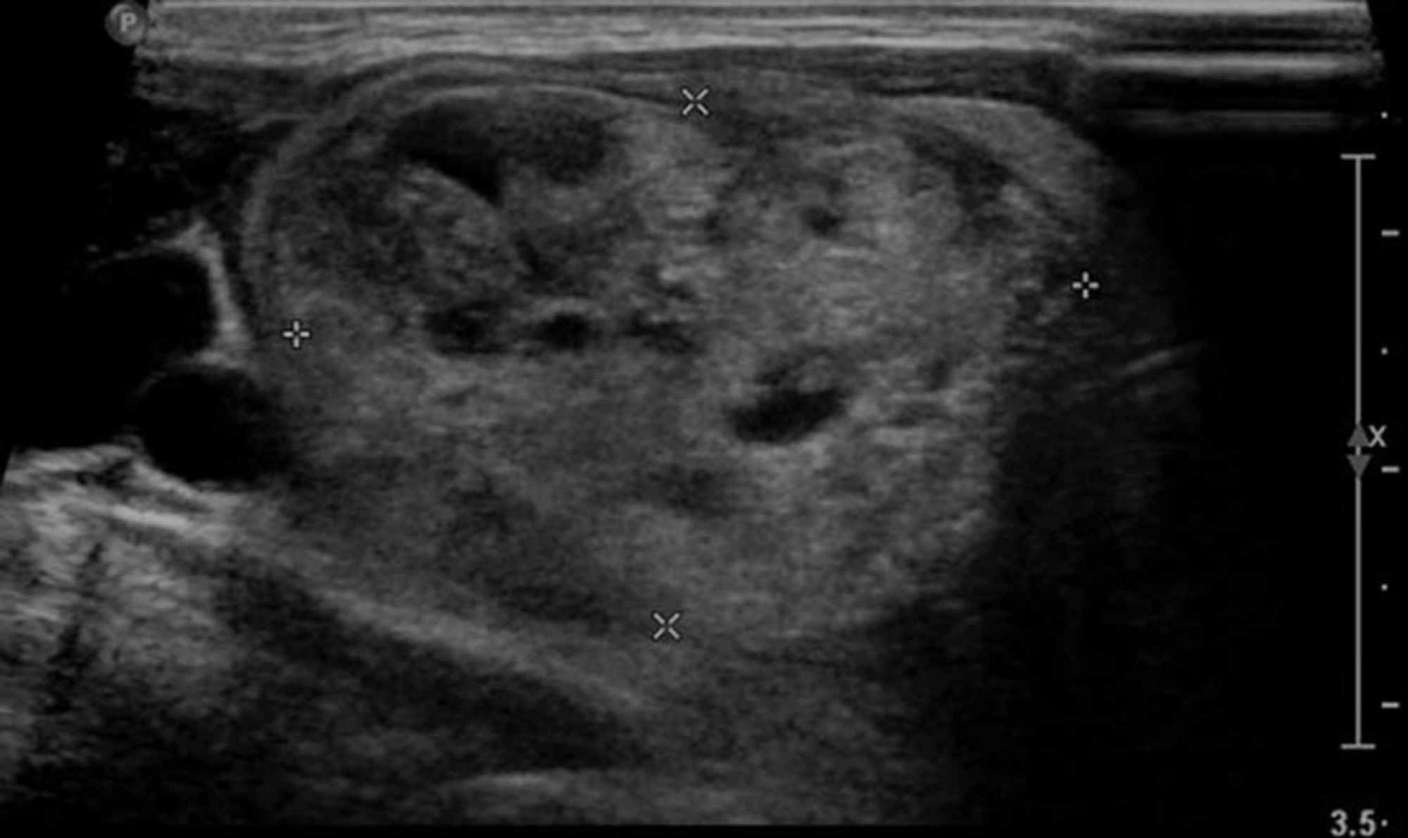
U/S of neck multinodular goiter in the right side measuring 3.2 x 2.4 cm U/S, ultrasound.

**Figure 4:**
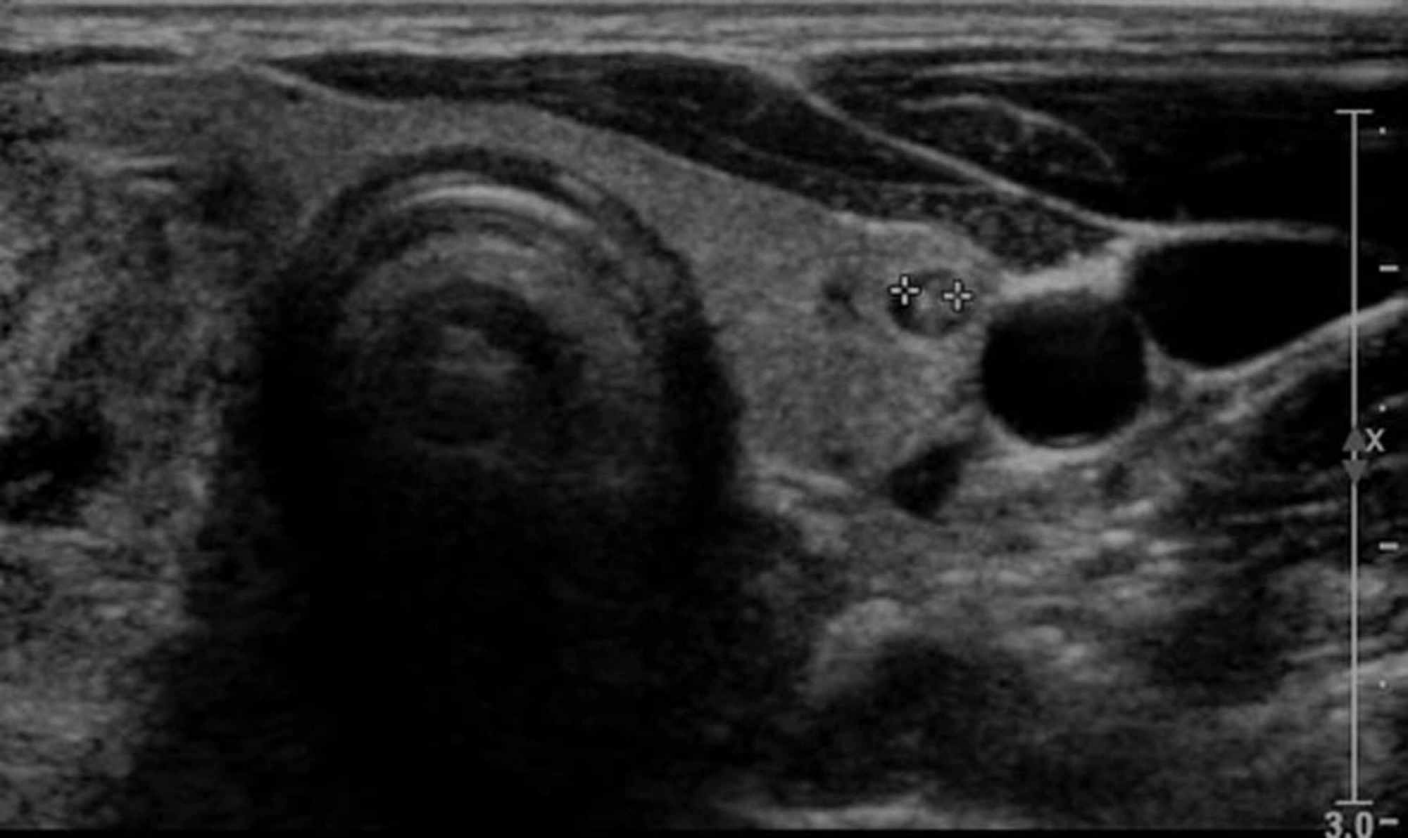
U/S of neck multinodular goiter in left side measuring 0.4 x 0.2 cm U/S, ultrasound.

Moreover, her Echo/Doppler cardiography report revealed grade I diastolic dysfunction and mild aortic regurgitation with normal left ventricular size and systolic function. Cardiac investigations were followed by thyroid profile where slightly low thyroid-stimulating hormone level was seen; however, T4 and T3 levels were normal. This was because she was kept on medication with adjustment made in dosages to achieve euthyroid levels. Later upon subsequent ultrasound of neck, multinodular goiter in the right side of 3.2 x 2.4 cm (Figure 3) and in the left side 0.4 x 0.2 cm (Figure 4) was observed. 

The patient was prepared for coronary artery bypass grafting (CABG) with pre-operative assessment showing a euthyroid state. After surgery, she was shifted to intensive care unit and was discharged a week with no post-operative complications. A follow-up of six months was recommended to assess the patient condition.

## Discussion

From BMS to the development of the second-generation DES, coronary stents have undergone a remarkable transformation with the aim to avoid both restenosis and thrombosis. The risk, however, is still present attributed to the stent being perceived as foreign prosthesis and subsequent neointima proliferation as the likely underlying pathology [8]. Variable investigations have looked into the predictors of ISR and recognized small vessel size, stents of increased length, diabetes mellitus, history of bypass surgery, and complex looking lesion as some of the metrics [9]

Although DES have been in Pakistan since 2002, few pieces of research have looked into the phenomenon and have been mostly centered around possible interventions like drug-eluting balloon and bioresorbable vascular scaffolds in the wake of ISR [10]. To the best of our knowledge, only a case of a late ISR was reported in October 2005 in the Pakistani literature and just a single investigation listing hypertension, diabetes mellitus, positive family history, dyslipidemia, and smoking as some of the triggering factors [11]. Even though the presentation of thyroid dysfunction as multinodular goiter is not that uncommon, the coexistence of multinodular goiter and its influence on the sequelae of coronary heart disease have never been emphasized in prior publications in Pakistan. Our case is, therefore, distinctive drawing attention to the comorbidity of multinodular goiter in ISR patients in the local population and the role played by the clinical parameter of hyperthyroidism on the outcome of the percutaneous intervention. To this effect, computed tomography angiography findings of patients with overt and subclinical hyperthyroidism had revealed a greater extent of high-grade and overall coronary stenoses, plaque burden, and high-risk plaque hallmarks than the euthyroid patients [12]. The data from Canpolat et al. substantiate this point, whereby the preprocedural serum level of fT4 was found to be a significant independent factor of ISR in patients with successful BMS implants [13].

Data on the overall epidemiology of MNG came up vague on our literature search; however, a study on histopathological findings of 624 thyroidectomies in Holy Family Hospital, Rawalpindi, Pakistan, did corroborate that MNG is the most common non-neoplastic lesion, with presenting age of patients ranging from 10 to 90 years. This emphasizes the importance of its cardiovascular implications, particularly in older patients who also have age as a non-modifiable risk factor [14].

ISR, on its own, is a nightmare for interventional cardiologists, with ongoing measures and precaution to circumvent this complication. With concurrent MNG and hyperthyroidism, it, without saying, puts the patient under a greater risk of ISR with high relevance in routine clinical practice. A close follow-up in such patients is mandatory. Our patient after primary percutaneous coronary intervention was lost to follow-up for six months after which she later presented with not only ISR but also the involvement of left circumflex and right coronary artery, thus progressing to three-vessel CAD with surgical revascularization through CABG as a reasonable option.

Here we want to highlight that while sifting literature we came across case reports where concomitant thyroidectomy was coupled with CABG with improved post-surgical outcomes, in addition to saving the patient from a second surgical intervention [15]. That being said, this was not a plausible option in our set up with its limited resources. In any event, this multidisciplinary approach should be kept in mind and given due consideration should the technical expertise and resources are available for surgical execution.

## Conclusions

Herein, we report a case of ISR in a known hyperthyroid patient. It is crucial to know any associations of altered thyroid function in the development of stent failures, be it in the form of ISR or stent thrombosis. Studies evaluating this relationship are meagre. Moreover, stent failure presents as a medical emergency; thus, it is essential for the cardiologists to know the triggers which cause it so as to maximize the efficacy of these tubes. 
